# Hypoglycemic Drugs in Patients with Diabetes Mellitus and Heart Failure: A Narrative Review

**DOI:** 10.3390/medicina60060912

**Published:** 2024-05-30

**Authors:** Anastasia Nikolaidou, Ioannis Ventoulis, Georgios Karakoulidis, Vasileios Anastasiou, Stylianos Daios, Spyridon-Filippos Papadopoulos, Matthaios Didagelos, John Parissis, Theodoros Karamitsos, Kalliopi Kotsa, Antonios Ziakas, Vasileios Kamperidis

**Affiliations:** 1 1st Department of Cardiology, AHEPA University Hospital, School of Medicine, Faculty of Health Sciences, Aristotle University of Thessaloniki, 54124 Thessaloniki, Greece; natasa_nikol@hotmail.com (A.N.); karakoulidisg@gmail.com (G.K.); vasianas44@gmail.com (V.A.); stylianoschrys.daios@gmail.com (S.D.); papadop00@yahoo.gr (S.-F.P.); manthosdid@yahoo.gr (M.D.); karamits@gmail.com (T.K.); tonyziakas@hotmail.com (A.Z.); 2 Department of Occupational Therapy, University of Western Macedonia, Keptse Area, 50200 Ptolemaida, Greece; iventoulis@uowm.gr; 3 Emergency Medicine Department, Attikon University Hospital, National and Kapodistrian University of Athens, Rimini 1, Chaidari, 10679 Athens, Greece; jparissis@yahoo.com; 4 Division of Endocrinology and Metabolism, Diabetes Center, 1st Department of Internal Medicine, AHEPA University Hospital, School of Medicine, Aristotle University of Thessaloniki, 54636 Thessaloniki, Greece; kkalli@auth.gr

**Keywords:** hypoglycemic drugs, diabetes mellitus, heart failure, cardiovascular outcomes

## Abstract

Over the last few years, given the increase in the incidence and prevalence of both type 2 diabetes mellitus (T2DM) and heart failure (HF), it became crucial to develop guidelines for the optimal preventive and treatment strategies for individuals facing these coexisting conditions. In patients aged over 65, HF hospitalization stands out as the predominant reason for hospital admissions, with their prognosis being associated with the presence or absence of T2DM. Historically, certain classes of glucose-lowering drugs, such as thiazolidinediones (rosiglitazone), raised concerns due to an observed increased risk of myocardial infarction (MI) and cardiovascular (CV)-related mortality. In response to these concerns, regulatory agencies started requiring CV outcome trials for all novel antidiabetic agents [i.e., dipeptidyl peptidase-4 inhibitors (DPP-4 inhibitors), glucagon-like peptide-1 receptor agonists (GLP-1 RAs), and sodium-glucose cotransporter-2 inhibitors (SGLT2is)] with the aim to assess the CV safety of these drugs beyond glycemic control. This narrative review aims to address the current knowledge about the impact of glucose-lowering agents used in T2DM on HF prevention, prognosis, and outcome.

## 1. Introduction

Ischemic heart disease is the leading cause of heart failure (HF) globally, particularly in heart failure with reduced ejection fraction (HFrEF) cases, while heart failure with preserved ejection fraction (HFpEF) is rising and associated with comorbidities like hypertension, obesity, chronic renal failure, coronary artery disease (CAD), older age, and type 2 diabetes mellitus (T2DM). T2DM is a chronic, complex disorder characterized by chronic hyperglycemia, which progressively leads to macrovascular (CAD, cerebrovascular, and peripheral vascular disease) and microvascular (nephropathy, retinopathy, and neuropathy) complications, increasing morbidity and mortality rates [1,2].

Additionally, the coexistence of T2DM and HF significantly increases mortality and hospitalization rates due to HF [1,3]. T2DM contributes to diabetic cardiomyopathy through various mechanisms. Hyperglycemia causes the glycation of proteins and lipids, forming advanced glycation end-products (AGEs) that stiffen the blood vessels and the myocardium, and impairs endothelial function, reducing nitric oxide availability, which increases vascular resistance. Furthermore, insulin resistance disrupts glucose and lipid metabolism, leading to fatty acid accumulation, mitochondrial dysfunction, lipotoxicity, and oxidative stress. Diabetes also triggers chronic systemic inflammation, causing myocardial inflammation, fibrosis, and apoptosis. Diabetic autonomic neuropathy affects baroreflex sensitivity and heart rate variability, while microvascular disease reduces coronary blood flow. Additionally, diabetic nephropathy leads to fluid retention and increased blood pressure, endothelial dysfunction, atherogenic dyslipidemia, and ultimately ischemia. These processes, through multiple pathways, lead to HF [2,4,5,6]. 

Historically, glucose-lowering drugs were chosen based on achieving optimal HbA1C levels (<53 mmol/mol or <7%), disregarding comorbidities [7]. However, recent emphasis has shifted towards cardiovascular (CV), renal, and mortality benefits. Since CV risks are associated with rosiglitazone, newer antidiabetic agents undergo rigorous CV safety evaluations through cardiovascular outcome trials (CVOTs). These trials mainly focus on 3-point major adverse cardiovascular events (3P-MACE) including CV death, nonfatal stroke, and nonfatal myocardial infarction (MI), with some including unstable angina (4P-MACE). In recent years, dipeptidyl peptidase-4 (DPP-4) inhibitors, glucagon-like peptide-1 receptor agonists (GLP-1 RAs), and sodium-glucose cotransporter-2 inhibitors (SGLT2is) have emerged as newer classes of antidiabetic agents, undergoing extensive testing for their CV effects and demonstrating promising outcomes in reducing CV risks among individuals with T2DM [8,9,10,11,12].

**Figure 1:**
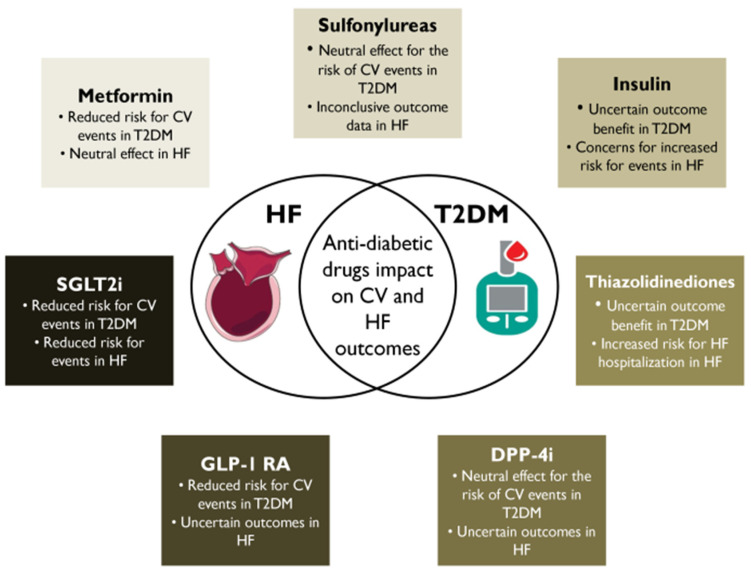
Antidiabetic drugs’ impact on cardiovascular and heart failure outcomes. CV, cardiovascular; HF, heart failure; T2DM, type 2 diabetes mellitus; DPP-4i, dipeptidyl peptidase-4 inhibitor; GLP-1 RA, glucagon-like peptide-1 receptor agonist; SGLT2i, sodium-glucose cotransporter 2 inhibitor.

This review focuses on the impact of classical and novel glucose-lowering agents on HF prevention and treatment, in patients with T2DM (Figure 1); the main studies providing evidence on the impact of each drug on 3P-MACE, HF hospitalizations, and cardiac function are analyzed.

## 2. Metformin

### 2.1. Clinical Cardiac Impact

Metformin is an effective low-cost antidiabetic drug, which has remained the first-line treatment for patients with T2DM since 2005, as long as there are no contraindications [13,14,15]. Predominantly concentrated in the liver, metformin acts in hepatocytes, especially in mitochondria, where it reduces the synthesis of adenosine triphosphate (ATP), leading to an increase in AMP-activated protein kinase, activating the AMPK pathway. This, in turn, inhibits hepatic gluconeogenesis and improves insulin sensitivity. In the intestine, metformin enhances incretin receptors and GLP-1 release in the plasma. Improvement in insulin sensitivity is also achieved by reducing lipogenesis in adipose tissue and increasing glucose uptake by the muscles, especially in the myocardium, through the AMPK pathway [14,16].

Historically, metformin faced contraindications for HF in 1977 due to a non-significant risk of lactic acidosis linked to phenformin [17]. However, based on subsequent observational studies and meta-analyses demonstrating a reduction in all-cause mortality, the Food and Drug Administration (FDA) reapproved metformin for HF in 2006 [13]. A large observational study in 2005 involving 12,272 individuals noted lower morbidity and mortality rates in T2DM and HF patients receiving metformin. Adjusted for covariates, metformin, either alone {adjusted hazard ratio (HR) 0.66, [95% confidence interval (CI) 0.44–0.97]} or in combination with other antidiabetic drugs [adjusted HR 0.54, (95% CI 0.42–0.70)], was associated with reduced 1-year mortality compared with sulfonylureas (SUs) [18].

In 2010, a small retrospective cohort study (242 subjects) suggested that metformin could enhance left diastolic myocardial function via improvements in two left ventricular (LV) diastolic parameters, a lower isovolumic relaxation time, and a higher early diastolic (e′) mitral annular velocity, in T2DM and HF or CAD patients. Based on experimental evidence from animal testing, there are indications that metformin may improve cardiac energy metabolism by stimulating AMP kinase (increases ATP via catabolic pathways). Additionally, it appears to enhance myocardial microcirculation, reducing ventricular stiffness and inhibiting cardiac fibrosis [19]. In the MET-REMODEL (Metformin and its Effects on Myocardial Dimension and Left ventricular hypertrophy in Normotensive patients with Coronary Heart Disease) trial including a small number of patients with CAD with prediabetes, after 12 months of treatment, metformin significantly reduced LV mass compared with placebo, indicating that metformin could be able to regress LV hypertrophy (LVH), an independent marker of CV events [20].

A meta-analysis of 11 observational studies demonstrated a 22% lower risk of all-cause mortality and a 13% lower risk of HF hospitalization among T2DM and HF patients treated with metformin compared to other glucose-lowering drugs [21].

The different impact of metformin on HFpEF and HFrEF was studied by a meta-analysis, involving 22,469 HF patients, that demonstrated a significant decrease in mortality rates in the HFpEF group treated with metformin, regardless of other cardio-protective treatments, while the impact on the HFrEF group was not statistically significant [14].

### 2.2. Clinical Decision-Making

Metformin is acknowledged as an essential first-line treatment for T2DM, with potential benefits to MACE and a neutral effect on HF. Based on observational studies, metformin demonstrated safety compared with insulin or sulfonylureas, but it is not recommended in patients with an estimated Glomerular Filtration Rate (eGFR) < 30 mL/min per 1.73 m^2^ [7].

## 3. SUs

### 3.1. Clinical Cardiac Impact

SUs are highly effective glucose-lowering agents and are in a heterogeneous antidiabetic drugs category, including various generations. So, as far as SU CV safety is concerned, it would be appropriate to distinguish them. Particularly, SUs are attached to receptors located within the plasma membrane of β-cells, which lead to membrane depolarization and consequently insulin release into the systemic circulation. This glucose-lowering action of SUs via persistent insulin secretion is glucose-independent, and hence, the major adverse event related to these drugs is hypoglycemia [16,22,23,24]. 

Many randomized controlled trials (RCTs) focus on new generation SUs, such as pancreas-specific glipizide and gliclazide, which bind reversible to their receptor acting short term. It was observed that glipizide and gliclazide were related to lower rates of in-hospital mortality in patients with MI in contrast to pancreas-nonspecific SUs [25]. Meanwhile, glyburide and glibenclamide, included in pancreas-nonspecific, long-acting SUs, inhibit ischemic preconditioning and may be related to an increased risk for MI [25,26]. These results were consistent with the outcome of a network meta-analysis of 167,327 patients, indicating that the new generation of SUs (glimepiride, gliclazide) are related to a lower risk of all-cause and CV mortality [27]. Among all SUs, gliclazide was associated with a decrease in the LV mass index in patients with T2DM, with a beneficial effect on LVH [28].

In the ADVANCE (Action in Diabetes and Vascular Disease: Preterax and Diamicron Modified Release Controlled Evaluation) trial, comprising 11,140 patients with T2DM randomized to standard glucose control (with no SUs) or intensive glucose control with gliclazide (plus other medications), there was no evidence that intensive treatment with gliclazide increased 3P-MACE, and no difference in the incidence of HF hospitalization was observed between the two groups [HR: 5(95% Cl −14 to 21)] [29].

Nevertheless, few observational studies including patients with T2DM indicated an increased risk of HF hospitalization in patients treated with SUs compared to metformin [30,31]. However, non-inferiority in the primary composite 3P-MACE outcome and secondary outcome of HF hospitalization was proved in CVOTs including patients with T2DM at high CV risk, which compared SUs with either pioglitazone or DPP-4 inhibitors (linagliptin) [32,33,34].

Regarding patients with T2DM and HF, a large observational study with 12,272 subjects reported lower rates of morbidity and mortality in patients treated with the metformin and SU combination compared to SU monotherapy [17]. Years later, a nationwide retrospective cohort study with 10,920 patients and a maximal 10-year follow-up provided strong evidence suggesting that SUs are inferior to metformin in terms of all-cause mortality HR [0.85 (95% CI 0.75–0.98, *p* = 0.02)] among patients with T2DM and HF [35].

### 3.2. Clinical Decision-Making

The conflicting evidence concerning SUs reflects the cautious approach recommended in some medical guidelines regarding the use of SUs, especially in patients with T2DM and HF [7].

## 4. Insulin

### 4.1. Clinical Cardiac Impact

Insulin is a hormone produced by the pancreas that plays a crucial role in regulating blood sugar levels. Insulin has direct effects on the heart, beyond its role in glucose metabolism. It facilitates the uptake of glucose into cells, including myocardial cells, and influences cardiac contractility and vascular function. Furthermore, insulin has been suggested to have anti-inflammatory effects, and inflammation is a key factor in the progression of HF [16,36].

However, evidence regarding the impact of insulin on HF is conflicting. The ORIGIN (Outcome Reduction with an Initial Glargine Intervention) trial, which randomized 12,537 patients with prediabetes or T2DM to basal insulin or placebo in addition to conventional therapy, found no difference in CV outcomes, including HF hospitalization [HR 0.90 (95% CI 0.77–1.05)] [37]. While the ORIGIN trial found insulin glargine to be safe in terms of CV outcomes, observational studies have suggested an increased risk of HF with insulin therapy in patients with diabetes mellitus (DM). The observational CHARM (Candesartan in Heart Failure Assessment of Reduction in Mortality and Morbidity) study showed that patients with established HF treated with insulin had a significantly increased risk of the composite outcome of CV death and HF hospitalization and all-cause mortality [38]. Additionally, observational studies and sub-analyses of RCTs showed an increased risk of death in patients with DM and HF treated with insulin [39,40,41].

### 4.2. Clinical Decision-Making

Nevertheless, the administration of insulin is sometimes necessary for adequate glycemic control in patients with DM hospitalized for acute decompensated HF. Of note, other glucose-lowering agents with proven CV benefits should be preferred if adequate glycemic control could be achieved without insulin [7,42]. 

## 5. Thiazolidinediones (TZDs)

### 5.1. Clinical Cardiac Impact

TZDs are highly effective glucose-lowering, inexpensive drugs for the therapy of DM, and their “pleiotropic” actions are observable after 1 month of initiation. These drugs target nuclear receptor peroxisome proliferator-activated receptors γ (PPARs): PPARγ1—located in all tissues and PPARγ2—located in adipose tissue and the intestine. TZDs via PPARγ regulate transcriptions factors related to insulin resistance and inflammatory and lipid metabolism. Mainly, TZDs decrease free fatty acid concentration on plasma by increasing lipogenesis and glucose uptake in adipose tissue alongside lowering gluconeogenesis in the liver and increasing insulin sensitivity in muscle tissue. However, TZDs cause fluid retention (peripheral edema), which has been linked to the incidence of HF in patients with or without DM. This side effect could be moderated by lowering doses of TZDs or by combining with other drugs (SGLT2i, GLP1-RAs), which increase sodium excretion [16,43].

Rosiglitazone and pioglitazone are the currently available TZDs in many countries for the management of T2DM alone or as add-on therapy to other hypoglycemic agents or insulin [44]. Specifically, a meta-analysis of rosiglitazone in 2007 showed a significant increase in the risk of MI and death from CV causes [45], and since then, the FDA mandated the conducting of powerful CVOTs, to evaluate the CV safety of all novel glucose-lowering drugs [46]. Nevertheless, 2 years later, the RECORD (Rosiglitazone Evaluated for Cardiac Outcomes and Regulation of Glycaemia in Diabetes) trial, signified the non-inferiority of rosiglitazone in overall CV morbidity or mortality, with inconclusive data for MI and an increased incidence of HF. Based on this trial, the FDA removed restrictions for rosiglitazone, but hesitation, especially in Europe, remains [46,47].

On the other side, in the Pro-active (PROspective pioglitAzone Clinical Trial In macroVascular Events) trial, pioglitazone in patients with T2DM and macrovascular disease was associated with a lower composite outcome of 3P-MACE versus placebo [HR 0.84 (95% CI 0.72, 0.98)] but also with an increase in the incidence of HF [48]. The incidence of HF was not related to higher morbidity and mortality rates, as it was evaluated by a post hoc analysis of the Pro-active study [49]. 

In a meta-analysis including 20,191 patients with prediabetes or T2DM at high CV risk, an increased risk of congestive HF was observed in patients treated with TZDs versus placebo or other agents [relative risk RR 1.72 (95% CI 1.21–2.42)] but no significant increase in CV death [RR 0.93 (95% CI 0.67–1.29)] [50]. Furthermore, many RCTs tried to evaluate the effect of pioglitazone on LV systolic and diastolic function in patients with T2DM, and the results were controversial. Few RCTs have demonstrated that pioglitazone is not associated with pivotal changes in LV function [51,52]. On the contrary, data from other RCTs suggested that treatment with pioglitazone in patients with T2DM increases left ventricular ejection fraction (LVEF) and stroke volume [53,54]. Of note, a recent RCT with 73 patients with T2DM and nonalcoholic fatty liver disease (NAFLD) found that pioglitazone improves global longitudinal strain (GLS), and NAFLD is a disease which is associated with an increased risk of developing HFpEF [55]. Although pioglitazone is recommended for patients with T2DM and NAFLD and based on animal model trials, reduces LV fibrosis, the potential role of this agent in HFpEF needs further investigation, especially because of the increase in the incidence of HF [56].

### 5.2. Clinical Decision-Making

Derived from these results, TZDs are generally contraindicated in patients with HF, and only pioglitazone is indicated in patients with established or at very high risk for CV disease, as second-line therapy with other agents with proven CV benefits [7]. 

## 6. DPP-4 Inhibitors

### 6.1. Clinical Cardiac Impact

DPP-4 inhibitors are oral glucose-lowering agents, which mimic and enhance the incretin effect, which is decreased in patients with T2DM. The incretin effect, which was first described in the early 1900s, answers to an increased insulin release via the oral administration of glucose, compared to intravenous, and it was mainly attributed to incretine hormones. Glucagon-like peptide-1 (GLP-1) and glucose-dependent insulinotropic polypeptide (GIP) are the major incretine hormones, which are produced, as a food intake answer, in the intestinal lumen and stimulate insulin secretion. The approach to amplify the incretin effect was either by GLP-1 RAs, with longer half-time, or by inhibitors of DPP-4, an enzyme responsible for the inactivation of incretin hormones [16,57]. 

Saxagliptin, alogliptin, sitagliptin, and linagliptin are recommended for the treatment of T2DM as alternative options after metformin. These oral hypoglycemic drugs are FDA-approved based on the results of CVOTs (Table 1), which have manifested CV safety but no CV risk reduction [7,58].

The SAVOR-TIMI 53 [Saxagliptin Assessment of Vascular Outcomes Recorded in Patients with Diabetes Mellitus (SAVOR)–Thrombolysis in Myocardial Infarction (TIMI) 53] study tried to evaluate the effect of saxagliptin on patients with T2DM at high risk for CV disease versus placebo. While the SAVOR-TIMI 53 trial demonstrated the safety of saxagliptin in the composite outcome of 3P-MACE, an unexpected increase in the risk of HF hospitalization was also noticed [HR, 1.27 (95% CI, 1.07–1.51)] [59]. Derived from these results, saxagliptin is contraindicated in patients with T2DM and HF [7,60]. Interestingly, an observational cohort including a few patients with T2DM did not observe any detrimental change in LV function or structure, as assessed by cardiac magnetic resonance imaging (CMR), when saxagliptin was added in the conventional treatment for 6 months [61].

Following large CVOTs, comparing DPP-4 inhibitors in patients with T2DM with or without established CV disease, with placebo or even with SUs, has indicated a neutral effect of these agents on CV death, risk for MI, urgent coronary revascularization, stroke, and HF hospitalization [33,34,62,63]. The results of the six major DPP-4 inhibitor trials were evaluated and enhanced by a meta-analysis including 52,520 patients with T2DM. According to the meta-analysis’s assessment, these hypoglycemic drugs represent a CV safe option for the management of T2DM but with a warning in the use of saxagliptin in HF. Major adverse events were not observed except for an increased risk of atrial flutter [64].

**Table 1:** Impact of dipeptidyl peptidase-4 inhibitors on heart failure hospitalization and cardiovascular death risk in cardiovascular outcome trials.

Study	Year	Drug	N	Baseline HF (%)	Baseline CVD (%)	Median Follow-Up (Years)	HF Hospitalization Risk [HR (95%CI), *p* Value]	CV Death Risk [HR (95%CI), *p* Value]
SAVOR-TIMI 53 [59]	2013	Saxagliptin	16,492	12.8	78	2.1	1.27 (1.07–1.51) 0.007	1.03 (0.87–1.22) 0.72
EXAMINE [63]	2013	Alogliptin	5,380	28	100	1.5	1.07 (0.79–1.46)	0.79 (0.60–1.04) 0.10
TECOS [62]	2015	Sitagliptin	14,671	18	100	3	1.00 (0.83–1.20) 0.98	1.03 (0.89–1.19) 0.71
CARMELINA [33]	2019	Linagliptin	6991	27	58	2.2	0.90 (0.74–1.08) 0.26	0.96 (0.81–1.14) 0.63
CAROLINA * [34]	2019	Linagliptin	6033	4.5	42	6.3	1.21 (0.92–1.59)	1.00 (0.81–1.24)

* Compared with glimepiride instead of placebo, N number, HF heart failure, CVD cardiovascular disease, HR hazard ratio, CI confidence interval.

Remarkably, a recent observational study with 2999 patients with T2DM and HF hospitalized in Japan has demonstrated that patients with HFpEF treated with DPP-4 inhibitors had a better HF hospitalization outcome compared to patients with heart failure with mild reduced ejection fraction (HFmrEF) or HFrEF [65].

LV diastolic and/or systolic function in patients with T2DM receiving DPP-4 inhibitors were evaluated by many RCTs. The addition of sitagliptin to the treatment regimens of patients with T2DM was associated with improvement in diastolic echocardiographic parameters (E/e′), indicating a potential beneficial effect on diastolic function [66], and regarding patients with T2DM and CAD, the addition of sitagliptin improved myocardial performance during stress echocardiography with dobutamine [67]. Meanwhile, adding linagliptin to the conventional treatment of patients with T2DM, concentric left ventricular geometry, and impaired systolic function was related to an insignificant increase in LV systolic function; the increase was higher in patients with worse echocardiographic abnormalities at baseline, indicating a potential benefit of linagliptin in this sub-patient’s category [68]. Furthermore, the VIVIDD (Vildagliptin in Ventricular Dysfunction Diabetes) trial remarked that vildagliptin versus placebo, after 52 weeks of treatment in patients with T2DM and HFrEF, resulted in increased left ventricular volumes but with a neutral effect on LVEF [69]. 

### 6.2. Clinical Decision-Making

**Figure 2:**
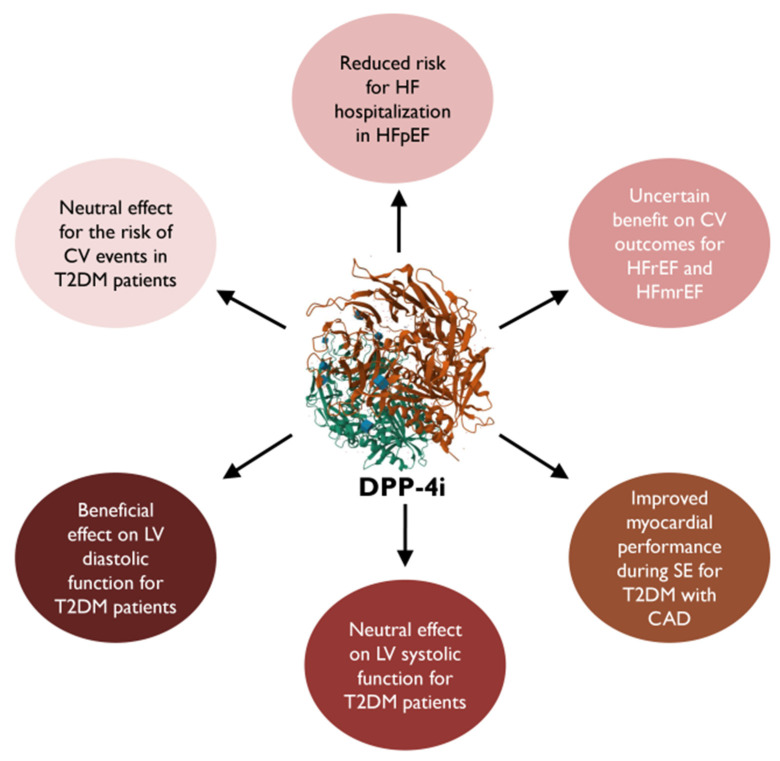
Dipeptidyl peptidase-4 inhibitors’ impact on cardiovascular and heart failure outcomes [70]. DPP-4i, dipeptidyl peptidase-4 inhibitors CV, cardiovascular; HF, heart failure; LV, left ventricular; T2DM, type 2 diabetes mellitus; HFpEF, heart failure with preserved ejection fraction; HFmrEF, heart failure with mild reduced ejection fraction; HFrEF, heart failure with reduced ejection fraction; SE, stress echocardiography; CAD, coronary artery disease.

In general, DPP-4 inhibitors are recommended in combination with other agents, targeting a holistic approach in the management of T2DM (Figure 2) [70], and concerning patients with established cardiovascular disease (CVD) or HF, these drugs could only be considered if the newer antidiabetic drugs (GLP-1 RAs, SGLT2i), which provide direct CV benefits, are contraindicated or not tolerated [7,9,42].

## 7. GLP-1 RAs

### 7.1. Clinical Cardiac Impact

GLP-1 RAs are highly effective antidiabetic drugs, which also enhance the incretin effect sturdy to DPP-4 metabolism and with a prolonged half-life time in plasma [71]. In particular, GLP-1 reduces hyperglycemia and stimulates insulin release in a glucose-dependent manner through many mechanisms, but more prominent are the suppression of glucagon release by pancreatic α-cells, the promotion of pancreatic β-cell proliferation and reduction in β-cell apoptosis, and the deceleration of gastric emptying [16]. The GLP-1 RAs approved by the FDA are subcutaneous exenatide twice-daily, lixisenatide once-daily, exenatide extended-release once-weekly, liraglutide once-daily, dulaglutide once-weekly, semaglutide once-weekly, and oral semaglutide once-daily [13,72,73].

GLP1-RAs have demonstrated safety in seven large CVOTs (Table 2) and based on the results of several meta-analyses, have also indicated a significant decrease in 3P-MACE and its individual components [9,74]. Of note, this beneficial effect was independent of baseline metformin treatment, and the exact mechanism by which some of these agents reduce CV outcome remains unclear [75]. GLP1-RA superiority in the reduction in 3P-MACE was also noticed in a network meta-analysis comparing GLP-1RAs to DPP-4 or SUs [76,77,78].

The LEADER (Liraglutide and Cardiovascular Outcomes in Type 2 Diabetes) trial was the first CVOT comparing liraglutide versus placebo in patients with T2DM irrespectively of their HF status, which showed an important reduction in 3P-MACE [HR 0.87 (95% CI 0.78–0.97)]. Specifically, liraglutide decreased rates of CV death and all-cause mortality, with a 22% and 15% reduction, respectively, and a neutral effect on HF hospitalization [79]. Additionally, in the SUSTAIN-6 (Trial to Evaluate Cardiovascular and Other Long-term Outcomes with Semaglutide in Subjects with Type 2 Diabetes) [80] and in REWIND (Researching cardiovascular Events with Weekly Incretin in Diabetes trial) trials, the rates of 3P-MACE were also lower compared with placebo, in patients with T2DM at high CV risk treated with semaglutide or dulaglutide, respectively [81]. In contrast to the LEADER trial, the results of the SUSTAIN-6 and REWIND trials were mainly attributed to statistical lower nonfatal stroke rates and not to lower mortality rates. The AMPLITUDE-O (Cardiovascular and renal outcomes with efpeglenatide in type 2 diabetes) trial evaluated the impact of another GLPI-RA in patients with T2DM with established or at risk for CVD versus placebo, and even though the study demonstrated a favorable effect on 3P-MACE, efpeglenatide is not yet FDA-approved [82].

Indeed, the majority of CVOTs have indicated the beneficial effect of GLP-1 on 3P-MACE, in patients with T2DM and established CVD from the perspective of secondary prevention. In addition, the REWIND trial, which included many patients with T2DM with no previous CVD or event, showed benefits to 3P-MACE in this subpopulation and demonstrated that dulaglutide might be effective even for primary prevention [79,80,81,82,83,84,85].

As far as HF is concerned, data from major GLP1-RA CVOTs indicated a neutral and potential beneficial effect on the relative risk of HF hospitalization, except from the SUSTAIN-6 trial, in which an increased risk of HF hospitalization was observed [79,80,81,82,83,84,85]. However, the results were insufficient, because quite a few patients with HF were included in these trials [86]. The HARMONY (Albiglutide and Cardiovascular Outcomes in Patients with Type 2 Diabetes and Cardiovascular Disease) trial was the only CVOT in which a GLP-1 RA was related to lower rates of HF hospitalization, but these reduced HF events were mainly noticed in patients without a previous history of HF [84,87].

Indeed, GLP1-RAs were analyzed in a recent meta-analysis, including 60,080 patients with T2DM at risk or with established CVD demonstrating not only a significant reduced risk of 3P-MACE and composite kidney outcome but also a lower risk of HF hospitalization [74,88]. 

Specifically, many RCTs tried to investigate the impact of GLP1-RAs οn LV diastolic and/or systolic function. An improved diastolic cardiac function in patients with T2DM was noticed after the initiation of exenatide, and compared to insulin glargine, exenatide improves both right ventricular global and LV regional subclinical dysfunction [89,90]. The MAGNA-VICTORIA (MAGNetic resonance Assessment of VICTOza efficacy in the Regression of cardiovascular dysfunction In type 2 diAbetes mellitus) trial investigated the impact of liraglutide on LV function, assessed by CMR, in patients with T2DM without CAD. Liraglutide decreased left ventricular filling pressures leading to left ventricular unload, verifying a positive effect on diastolic function and a neutral on systolic ventricular function [91]. A meta-analysis concluded that liraglutide was associated with a more significant improvement in LV diastolic function in patients with T2DM compared to the other GLP-1 RAs [92]. In addition, a recent meta-analysis of 22 RCTs evaluated the effect of GLP-1 RAs on cardiac function in patients with T2DM and based on the positive effect on diastolic echocardiographic parameters, early diastolic-to-late diastolic velocities ratio (E/A ratio), mitral inflow E velocity-to-tissue Doppler e′ ratio (E/e′ ratio), and E-wave deceleration time, demonstrated an improvement in left ventricular diastolic function by GLP-1 RAs [93]. 

Nevertheless, the FIGHT (Functional Impact of GLP-1 for Heart Failure Treatment) trial investigated the effect of liraglutide on patients with acute decompensated HF (median LVEF~25%) versus placebo. Liraglutide did not reduce the risk of HF hospitalization or death [HR 1.30 (95% CI 0.92–1.83)], and specifically in patients with T2DM, liraglutide was linked to higher rates of HF events [94]. Additionally, in a recent post hoc analysis of the FIGHT trial, the results were disappointing due to an increased risk of HF hospitalization and all-cause deaths associated with higher rates of arrhythmias, especially in patients with severely symptomatic HF [75]. Furthermore, the LIVE (Effect of Liraglutide, a Glucagon-like Peptide-1 Analogue, on Left Ventricular Function in Stable Chronic Heart Failure Patients With and Without Diabetes) trial randomized patients with LVEF < 45% and stable HF to liraglutide or placebo. However, after 24 weeks of initiation, the change in LVEF between the intervention and placebo group was not statistically significant; higher rates of arrhythmia and ischemic cardiac events were noticed in patients treated with liraglutide [95]. Indeed, the administration of liraglutide leads to an increased heart rate that could be linked to arrhythmias, but the exact mechanism by which liraglutide could lead to an increased risk of serious cardiac effects in patients with HFrEF remains unclear [75,95,96].

### 7.2. Clinical Decision-Making

**Figure 3:**
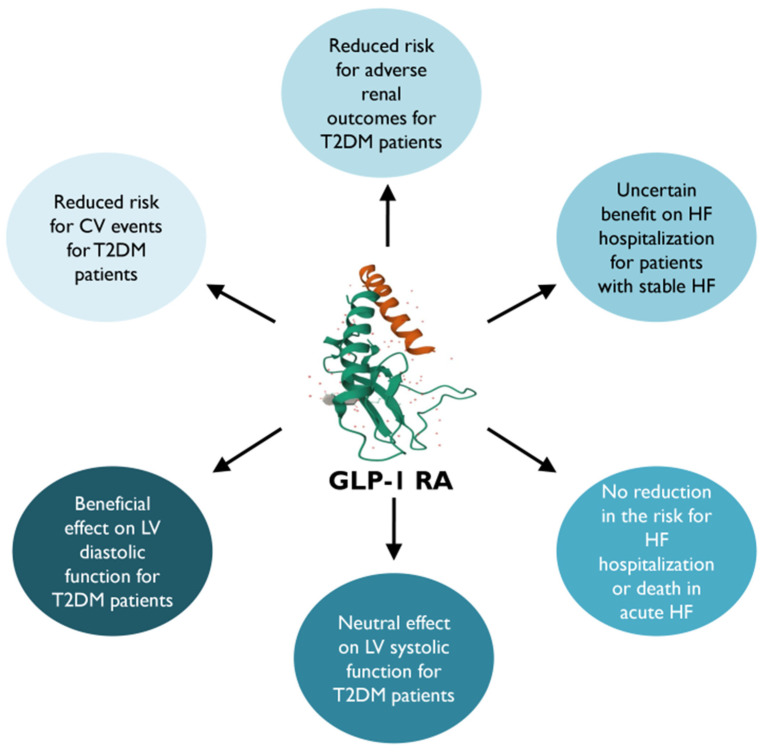
Glucagon-like peptide-1 receptor agonists’ impact on cardiovascular and heart failure outcomes [97]. GLP-1 RA, glucagon-like peptide-1 receptor agonist; CV, cardiovascular; HF, heart failure; T2DM, type 2 diabetes mellitus; LV, left ventricular.

In general, GLP-1 RAs have demonstrated a lower risk of 3P-MACE in patients with T2DM with multiple CV risk factors or established CVD with a more neutral effect on HF hospitalization (Figure 3) [9,97]. Meanwhile, most of the results suggest that in those with established HFrEF, treatment with GLP-1RAs may increase the risk of adverse effects. However, in those without HF history, GLP-1RAs may prevent the development of HF [75]. Ongoing research continues to explore the mechanisms underlying the CV benefits of this drug category.

## 8. SGLT2i

### 8.1. Clinical Cardiac Impact

SGLT2is, or gliflozins, are oral glucose-lowering drugs of great scientific interest, since recently they have shown their beneficial effect on major CV and renal outcomes, irrespectively of DM presence. SGLT2is achieve their intermediate-to-high hypoglycemic efficacy by targeting a different pathophysiological pathway compared to other hypoglycemic agents. In fact, gliflozins reduce plasma glucose in an insulin-independent manner by causing glucosuria via the inhibition of sodium-glucose cotransporters 2 (SGLT2s) in the kidneys [16,98].

### 8.2. Major CVOTs

In the first place, SGLT2is (dapagliflozin, empagliflozin, and canagliflozin) were approved by the FDA for the treatment of T2DM [13]. Subsequently, several clinical trials have indicated significant CV and renal beneficial effects of SGLT2is manifesting the revolutionary role of gliflozins (Table 3).

Specifically, the EMPA-REG OUTCOME (Empagliflozin, Cardiovascular Outcomes, and Mortality in Type 2 Diabetes) trial was the first published CVOT, which demonstrated the beneficial effect of empagliflozin on 3P-MACE and HF hospitalization in patients with T2DM and established CVD versus placebo. As it was indicated after a 3-year follow-up, empagliflozin in addition to standard therapy reduced 3P-MACE by 14%, all-cause mortality by 32%, CV mortality by 38%, and HF hospitalization by 35% [99]. 

In 2017, another trial randomized 10,142 patients with T2DM and established/high risk of CVD to canagliflozin or placebo, with a median observation time of 3.5 years. Although canagliflozin reduced 3P-MACE by 14% and HF hospitalization by 33%, the reduction in all-cause and CV mortality was not statistically significant [100]. Similar were the results in the DECLARE-TIMI 58 (The Dapagliflozin Effect on Cardiovascular Events–Thrombolysis in Myocardial Infarction 58) trial, which also recruited 17,160 patients with T2DM and at risk or with established CVD. After a 4.2-year follow-up, dapagliflozin was associated with a significantly lower risk of HF hospitalization [HR, 0.73 (95% CI, 0.61 to 0.88)] compared to placebo but failed to demonstrate significant reduction in the primary composite of 3P-MACE [HR, 0.93 (95% CI, 0.84 to 1.03)], all-cause and CV mortality [101,102]. Finally, the VERTIS (Cardiovascular Outcomes with Ertugliflozin in Type 2 Diabetes) trial evaluated the efficacy and safety of another recent FDA-approved SGLT2i. After a 3.5-year follow-up, ertugliflozin indicated a 30% lower risk of HF hospitalization but a neutral effect on 3P-MACE and its components, in patients with T2DM and established CVD [103]. 

In general, based on the results of major CVOTs including patients with T2DM, SGLT2is lead to a significant reduction in HF hospitalization in view of the secondary prevention of patients with established CVD, and only dapagliflozin in DECLARE-TIMI 58 also targeted primary prevention in individuals at high risk for CVD [101,102]. Indeed, the large observational CVD-REAL (Comparative Effectiveness of Cardiovascular Outcomes in New Users of Sodium-Glucose Cotransporter-2 Inhibitor) study, which included >300,000 individuals with T2DM across five countries, showed that, independently of pre-existing CVD, patients treated with SGLT2is compared to other antidiabetic drugs had a significantly lower risk of HF hospitalization and death [104].

**Table 3:** Impact of sodium-glucose cotransporter 2 inhibitors on hospitalization for heart failure and cardiovascular death risk in cardiovascular, renal, and heart failure outcome trials.

Study	Year	Drug	N	Baseline HF (%)	Baseline CVD (%)	Median Follow-Up (Years)	HF Hospitalization Risk [HR (95%CI), *p* Value]	CV Death Risk [HR (95%CI), *p* Value]
EMPA-REG [99]	2015	Empagliflozin	7020	10	99	3.1	0.65 (0.50–0.85) 0.002	0.62 (0.49–0.77) <0.001
CANVAS [100]	2017	Canagliflozin	10,142	14.4	65.6	2.4	0.67 (0.52–0.87)	0.87 (0.72–1.06)
DECLARE-TIMI 58 [101]	2018	Dapagliflozin	17,160	10	41	4.2	0.73 (0.61−0.88)	0.98 (0.82−1.17)
VERTIS-CV [103]	2020	Ertugliflozin	8246	23.7	75.9	3	0.70 (0.54–0.90)	0.92 (0.77–1.11)
DAPA-HF * [105]	2019	Dapagliflozin	4744	100	56	1.5	0.7 (0.59–0.83)	0.82 (0.69–0.98)
EMPEROR-reduced * [106]	2020	Empagliflozin	3730	100	52	1.3	0.69 (0.59–0.81)	0.92 (0.75–1.12)
SOLOIST-WHF [107]	2020	Sotagliflozin	1222	100	ΝA	0.75	0.64 (0.49 to 0.83) <0.001	0.84 (0.58 to 1.22) 0.36
EMPEROR-preserved * [108]	2021	Empagliflozin	5988	100	35.5	2.2	0.71 (0.60–0.83)	0.91 (0.76–1.09)
DELIVER [109]	2022	Dapagliflozin	6263	100	NA	2.3	0.79 (0.69–0.91)	0.88 (0.74–1.05)
CREDENCE [110]	2019	Canagliflozin	4401	14.8	50.4	2.62	0.61 (0.47–0.8) <0.001	0.78 (0.61–1.00) 0.05
SCORED [111]	2020	Sotagliflozin	10,584	31	22	1.3	0.67 (0.55–0.82) <0.001	0.90 (0.73–1.12) 0.35
DAPA-CKD * [112]	2020	Dapagliflozin	4304	11	37.5	2.4	0.71 (0.55–0.92) **	0.81 (0.58–1.12)

* Recruited patients with and without T2DM, ** composite outcome of heart failure hospitalization and cardiovascular mortality, N number, HF heart failure, CVD cardiovascular disease, HR hazard ratio, Cl confidence interval.

### 8.3. HF Outcome Trials

Based on the results of prior CVOTs, which have indicated the beneficial effect of SGLT2is on a subgroup population with HF and T2DM, two large trials were conducted to evaluate the effect of SGLT2is on HFrEF [105,106]. DAPA-HF (Dapagliflozin and Prevention of Adverse Outcomes in Heart Failure) recruited 4744 patients with HFrEF (LVEF < 40%), which were randomized to dapagliflozin or placebo, in addition to standard therapy, and the primary composite outcome was the worsening of HF or CV death. After 1.5 years of follow-up, patients treated with dapagliflozin had a significantly lower risk of the primary composite outcome [HR 0.74 (95% CI 0.65 to 0.85)] and worsening HF [HR 0.70 (95%Cl 0.59–0.83)], compared to the placebo group [105]. The design of the EMPEROR-Reduced (Empagliflozin Outcome Trial in Patients with Chronic Heart Failure with Reduced Ejection Fraction) trial was similar to DAPA-HF. Indeed, 3730 patients with HFrEF (LVEF < 40%) were randomized to receive empagliflozin or placebo, and as it was proven after 1.3 years of follow-up, empagliflozin reduced the primary outcome by 25% and HF hospitalization by 30%. These findings were consistent in patients with and without T2DM [106]. Although EMPEROR-Reduced showed a significant decrease in the risk of HF hospitalization, it failed to demonstrate a statistically significant reduction in all-cause and CV mortality, as it was demonstrated in DAPA-HF. This neutral effect of empagliflozin on mortality could be attributed to the design of EMPEROR-Reduced, which included patients with higher natriuretic peptide levels and lower LVEF (LVEF 27–31%) [101,106]. 

The favorable effects of empagliflozin on HFrEF were corroborated in patients with or without T2DM and acute decompensated HF or renal failure [107,108,109,110,111,112,113,114]. The EMPA-RESPONSE-AHF (Randomized, Double-blind, Placebo-controlled, Multicentre Pilot Study on the Effects of Empagliflozin on Clinical Outcomes in Patients with Acute Decompensated Heart Failure) trial showed a significant reduction in the composite HF outcome of in-hospital worsening HF, the rehospitalization of HF or death at 60 days after the initiation of empagliflozin compared with placebo [4 (10%) vs. 13 (33%); *p* = 0.014] [113]. Even at 90 days after administration, empagliflozin was associated with a sustained decongestion benefit and clinical improvement [114]. Additionally, the placebo-controlled SOLOIST-WHF (the Effect of Sotagliflozin on Cardiovascular Events in Patients With Type 2 Diabetes Post Worsening Heart Failure) trial demonstrated that, in patients with T2DM and worsening HF, sotagliflozin was associated with a reduced outcome of CV deaths or hospitalizations or urgent visits for HF [HR 0.67 (95% CI 0.52–0.85)] regardless of LVEF. Specifically, the early initiation of sotagliflozin during hospitalization resulted in lower CV and HF events at 30 and 90 days after discharge [107].

Additionally, in the EMPEROR-Preserved (Empagliflozin Outcome Trial in Patients with Chronic Heart Failure with Preserved Ejection Fraction) trial, empagliflozin was associated with a significantly lower risk of HF hospitalization [HR 0.73 (95% CI 0.61–0.88)] and the composite of worsening HF and CV death [HR 0.79 (95% CI 0.69–0.90)], in patients with HFmrEF and HFpEF (LVEF > 40%) [108]. Recently, the DELIVER (Dapagliflozin in Heart Failure with Mildly Reduced or Preserved Ejection Fraction) trial demonstrated that dapagliflozin in patients with LVEF > 40% could reduce the primary outcome by 18% and worsening HF by 21% [109]. The results were similar in patients with and without T2DM. Hence, SGLT2is (empagliflozin or dapagliflozin) are recommended (class IA) in patients with symptomatic HFmrEF or HFpEF alongside diuretics for fluid retention [10].

Generally, SGLT2is improve the composite outcome of worsening HF and CV death in patients with HF, irrespectively of LVEF or diabetes presence or established chronic kidney disease (CKD). Indeed, SGLT2is reduced each one of the composites independently, with a more pronounced reduction in the risk of HF hospitalization, additionally reducing the risk of adverse side effects. Although SGLT2is were not associated with significantly lower rates of all-cause mortality risk, there was a trend of reduction compared with placebo [115,116,117,118,119]. Furthermore, comparing with other antidiabetic drugs, SGLT2is established their superiority over DPP-4 inhibitors in lowering the risk of most composite cardio-renal endpoints and over GLP-1 RAs in reducing the risk for HF hospitalization [9].

### 8.4. Cardiac Function Impact

**Figure 4:**
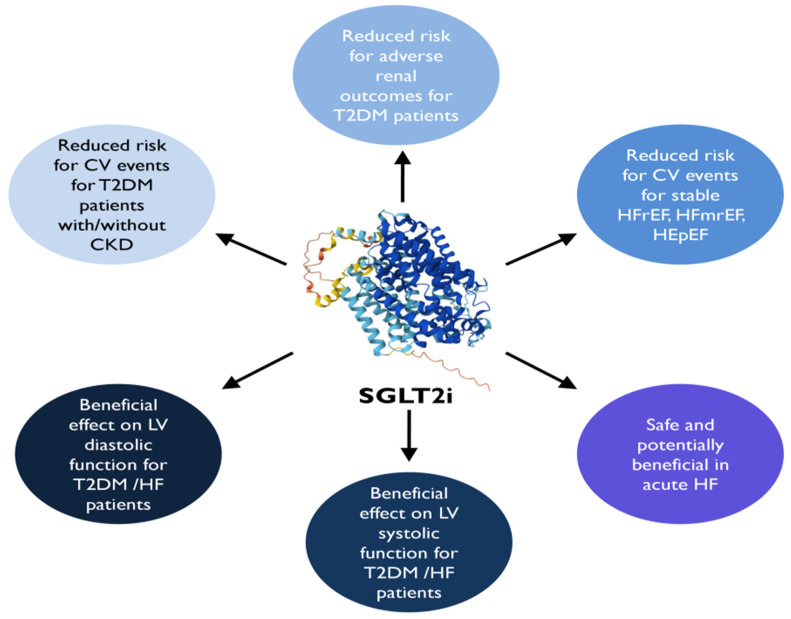
Sodium-glucose cotransporter-2 inhibitors’ impact on cardiovascular and heart failure outcomes [120]. SGLT2i, sodium-glucose cotransporter-2 inhibitor; CV, cardiovascular; HF, heart failure; T2DM, type 2 diabetes mellitus; HFpEF, heart failure with preserved ejection fraction; HFmrEF, heart failure with mild reduced ejection fraction; HFrEF, heart failure with reduced ejection fraction; LV, left ventricular; CKD, chronic kidney disease.

Several RCTs have subsequently focused on the impact of SGLT2is on cardiac function and structure, derived from the evident salutary effects of these agents on HF (Figure 4) [116,120]. In patients with T2DM and HFrEF, treatment with empagliflozin for 36 weeks reversed LV remodeling by reducing the LV end-diastolic and systolic volume index [121]. Specifically, the recently published EmDia (Effects of empagliflozin on left ventricular diastolic function in addition to usual care in individuals with type 2 diabetes mellitus) trial concluded that empagliflozin improved LV diastolic dysfunction in patients with T2DM, including those with HFpEF. After 12 weeks of empagliflozin initiation, the primary endpoint E/e′ ratio was lower [−1.18 (95% CI; −1.72 to −0.65] compared to placebo [122]. In addition, a significant LV mass reduction [−2.82 g (95% CI; −5.13 to −0.51)], after dapagliflozin initiation, was observed in patients with T2DM, LVH, and controlled blood pressure, demonstrating a positive effect of dapagliflozin on cardiac remodeling and LV structure [123]. Meanwhile, dapagliflozin in the REFORM (Dapagliflozin Versus Placebo on Left Ventricular Remodeling in Patients With Diabetes and Heart Failure) trial did not indicate a favorable effect on reversing LV remodeling in individuals with T2DM and HFrEF [124]. Recently, a meta-analysis was published evaluating the effect of SGLT2is on cardiac imaging parameters (assessed by CMR or echocardiography) in a diverse population. Of note, treatment with SGLT2is compared with placebo outlined a positive change in the left atrial volume index and E/e′ on imaging, demonstrating that improved LV diastolic function by SGLT2is could be associated with beneficial effects on HF outcome. Furthermore, a trend for reduction in LV mass and volume parameters was observed and an increase in LV function parameters (LVEF, LVGLS, and stroke volume) [125] with a more pronounced effect on HFrEF patients [126].

## 9. SGLT2i and GLP-RA Combination

Beyond doubt, both SGLT2is and GLP-RAs in major CVOTs have shown significant beneficial effects on CV and renal outcomes, mainly through glucose-lowering-independent and distinct mechanisms. Consequently, their combination in patients with T2DM and at risk for or with CVD could enhance their positive cardio-renal effects, through synergistic or complementary pathways [127].

### 9.1. Established Knowledge

A post hoc analysis of EXSCEL (Effects of Once-Weekly Exenatide on Cardiovascular Outcomes in Type 2 Diabetes) tried to evaluate the effect of SGLT2is plus GLP-1 RAs combined therapy, in patients with T2DM with or without CVD. The co-administration of exenatide and SGLT2i reduced 3P-MACE with a pronounced decrease in CV mortality, compared to exenatide alone and without increasing the risk of hypoglycemia [128]. The results from a large retrospective cohort, including ~2 million patients with T2DM, showed that combination therapy was related to a significantly lower risk of all-cause mortality compared to monotherapy [129]. In terms of myocardial function, dual treatment improved LV myocardial function markers, such as GLS, compared to SGLT2is, GLP-1 RAs, or insulin alone, particularly in individuals with HF and LVEF < 55% [130]. In addition, the combined SGLTi and GLP-1 RA regimen was associated with a lower risk of incident HF, compared to monotherapy or other antidiabetic combinations, demonstrating the effective primary prevention of HF [131].

In a recent meta-analysis of five CVOTs, dual therapy was associated with a significant reduction in 3P-MACE but similar to either SGLT2is or GLP-1 RAs alone. However, combination therapy was related to an added beneficial effect on lowering HF hospitalization compared to monotherapy [127].

### 9.2. Future Directions

To date, there have been no published CVOTs to evaluate the cardio-renal outcomes and mortality of these two drug classes’ combination. While HF outcome trials with the combined SGLT2i plus GLP-1 RA therapy are lacking, there are promising findings concerning the potential favorable effect of their combination on HFpEF [132]. In the case of HFrEF, there is a forewarning regarding GLP-1 RAs, based on the results of the FIGHT and LIVE trials, and therefore, the co-administration of SGLT2is and GLP-1 RAs requires awareness and an individualized approach [133]. 

## 10. Indication of Antidiabetic Drugs in HF According to Guidelines

**Figure 5:**
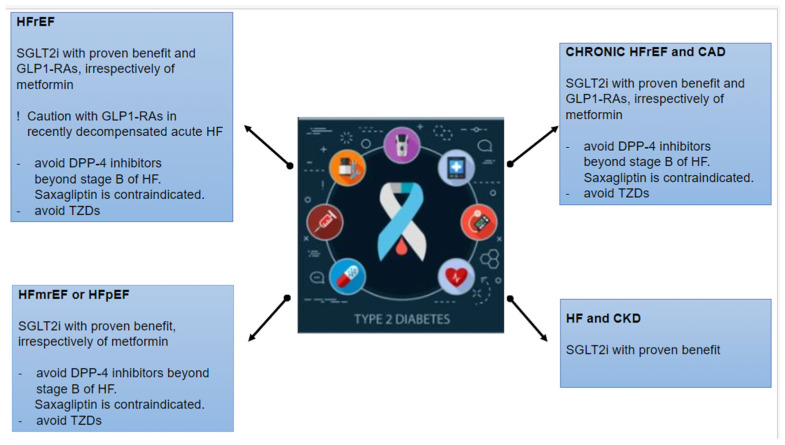
Indications of antidiabetic drugs in multiple heart failure phenotypes according to guidelines. CAD, coronary artery disease; HF, heart failure; DPP-4 inhibitors, dipeptidyl peptidase-4 inhibitors; GLP-1 RA, glucagon-like peptide-1 receptor agonist; SGLT2i, sodium-glucose cotransporter 2 inhibitor, TZDs, thiazolidinediones; HFpEF, heart failure with preserved ejection fraction; HFmrEF, heart failure with mild reduced ejection fraction; HFrEF, heart failure with reduced ejection fraction; CKD, chronic kidney disease.

The guidelines of ACC/AHA/HFSA (Heart failure Society of America), EASD (European Association for the Study of Diabetes)/ADA (American Diabetes Association)), and ESC concerning antidiabetic drugs in DM and HF indicate the following (Figure 5) [7,10,42,60,134]:

- **Metformin** remains the first-line treatment in combination with lifestyle interventions in patients with T2DM without cardio-renal comorbidities. In terms of CV and renal outcomes, novel antidiabetic agents (SGLT2is and GLP-1 RAs) have proven their beneficial effects on 3P-MACE, HF hospitalization, and mortality independent of metformin use. Hence, these drugs should be firstly considered in patients with established or a high risk of CVD, HF, and CKD, irrespectively of metformin use. In patients with T2DM and stable HF, metformin may be continued for glucose lowering if the eGFR remains > 30 mL/min/1.73 m^2^ but should be avoided in unstable or hospitalized individuals with HF.
- **SUs** have met controversial results concerning CV safety and the risk of HF hospitalization. Hence, in patients with T2DM and HF, SUs should only be considered in the case of poor glycemic control with alternative options and be used with caution.
- **Insulin** should be considered in patients with DM and acute decompensated HF.
- **TZDs:** Pioglitazone could be considered as second-line therapy in patients at very high risk or with established CVD, if the glycemic target is not achieved or novel agents are contraindicated. However, TZDs (pioglitazone and rosiglitazone) are generally contraindicated in patients with T2DM at risk or with established HF due to the increase in HF incidence.
- **DPP-4 inhibitors** (sitagliptin and linagliptin) showed a neutral effect on the risk of HF hospitalization or 3P-MACE and may be considered for DM management in patients with HF. Only saxagliptin was related to an increased risk of HF hospitalization, and it is not recommended in patients with T2DM at risk or with manifest HF. However, the AHA/ACCF/HFSA consensus recommends avoiding DPP-4 inhibitors over stage-B HF.
- **GLP-1 RAs** are highly recommended in patients with T2DM, with or without established CVD and irrespectively of other hypoglycemic therapies or glycose-lowering targets. Specifically, a GLP-1 RA with proven benefits could be used in patients with very high risk for CVD (>55 years, hypertension, smoking, dyslipidemia, obesity, or albuminuria) and should be used in patients with established CVD, to reduce 3P-MACE. GLP-1 RAs (lixisenatide, liraglutide, exenatide, semaglutide, and dulaglutide) had a neutral effect on HF hospitalization and could be considered as an alternative treatment of DM in patients with HF. However, it would be preferable to avoid GLP-1 RAs in HFrEF and recently decompensated acute HF.
- **SGLT2is** are recommended in patients with T2DM with or without established CVD, HF, or CKD (eGFR > 20 mL/min per 1.73 m²), to reduce 3P-MACE and improve kidney outcomes, irrespectively of other antidiabetic drugs or glucose-lowering goals. Specifically, dapagliflozin, empagliflozin, and sotagliflozin are strongly recommended in patients with T2DM and CVD or HF, irrespectively of LVEF, to reduce HF hospitalization and CV death.

## 11. Conclusions

In conclusion, patients with prediabetes or DM are at an increased risk of developing HFpEF or HFrEF, and concomitantly, HF increases the risk of developing T2DM. The prognosis for HF is notably more adverse in patients with compared to those without T2DM. Currently, antidiabetic medications emphasize not only effectively controlling blood glucose levels but also demonstrating CV safety. Novel antidiabetic drugs, SGLT-2is and GLP-1RAs, have shown impressive CV benefits, particularly in reducing HF hospitalization, CV death, and demonstrated beneficial effects on cardiac function. Overall, the selection of glucose-lowering agents should be individualized, considering personal preferences, encompassing comorbidities, protecting the kidneys’ function, reducing side effects, and mainly targeting optimal CV outcomes.

## Figures and Tables

**Table 2:** Impact of glucagon-like peptide-1 receptor agonists on hospitalization for heart failure and cardiovascular death risk in cardiovascular outcome trials.

Study	Year	Drug	N	Baseline HF (%)	Baseline CVD (%)	Median Follow-up (Years)	HF Hospitalization Risk [HR (95%CI), *p* Value]	CV Death Risk [HR (95%CI), *p* Value]
ELIXA [73]	2015	Lixisenatide	6068	22	100	2.1	0.96 (0.75–1.23)	0.98 (0.78 −1.22)
LEADER [79]	2016	Liraglutide	9340	17.8	81	3.8	0.87 (0.73–1.05)	0.78 (0.66–0.93)
SUSTAIN-6 [80]	2016	Semaglutide	3297	23.6	83	2.1	1.11 (0.77–1.61)	0.98 (0.65–1.48)
REWIND [81]	2019	Dulaglutide	9901	8.6	31.5	5.4	0.93 (0.77-1.12)	0.91(0.78-1.06)
AMPLITUDE-O [82]	2021	Efpeglenatide	4076	18.1	89.6	1.81	0.61 (0.38–0.98)	0.72 (0.50–1.03)
EXSCEL [83]	2017	Exenatide	14,752	16	73.1	3.2	0.94 (0.78−1.13)	0.88 (0.76−1.02)
HARMONY [84]	2018	Albiglutide	9463	20	100	1.6	0.71 (0.53–0.94)	0.93 (0.73–1.19)
PIONEER-6 [85]	2019	Semaglutide	3183	NA	85	1.3	0.86 (0.48–1.55)	0.49 (0.27–0.92)

N number, HF heart failure, HR hazard ratio, NA not applicable.
